# Fertility treatments and the risk of preterm birth among women with subfertility: a linked-data retrospective cohort study

**DOI:** 10.1186/s12978-022-01363-4

**Published:** 2022-03-29

**Authors:** Jessica N. Sanders, Sara E. Simonsen, Christina A. Porucznik, Ahmad O. Hammoud, Ken R. Smith, Joseph B. Stanford

**Affiliations:** 1 Department of Obstetrics and Gynecology, University of Utah, S30 N 1900 E, Rm 2B200, Salt Lake City, UT 84132 USA; 2 Department of Family and Preventive Medicine, University of Utah School of Medicine, 375 Chipeta Way Ste A., Salt Lake City, UT 84108 USA; 3 University of Utah College of Nursing, 10 South 2000 East, Salt Lake City, UT 84112 USA; 4 University of Utah Pedigree and Population Resource (Utah Population Database), Population Sciences, Huntsman Cancer Institute, 2000 Circle of Hope, Salt Lake City, UT 84112 USA

**Keywords:** Infertility, Pregnancy, Preterm birth, Fertilization in vitro

## Abstract

**Background:**

In vitro fertilization (IVF) births contribute to a considerable proportion of preterm birth (PTB) each year. However, there is no formal surveillance of adverse perinatal outcomes for less invasive fertility treatments. The study objective was to describe associations between fertility treatment (in vitro fertilization, intrauterine insemination, usually with ovulation drugs (IUI), or ovulation drugs alone) and preterm birth, compared to no treatment in subfertile women.

**Methods:**

The Fertility Experiences Study (FES) is a retrospective cohort study conducted at the University of Utah between April 2010 and September 2012. Women with a history of primary subfertility self-reported treatment data via survey and interviews. Participant data were linked to birth certificates and fetal death records to asses for perinatal outcomes, particularly preterm birth.

**Results:**

A total 487 birth certificates and 3 fetal death records were linked as first births for study participants who completed questionnaires. Among linked births, 19% had a PTB. After adjustment for maternal age, paternal age, maternal education, annual income, religious affiliation, female or male fertility diagnosis, and duration of subfertility, the odds ratios and 95% confidence intervals (CI) for PTB were 2.17 (CI 0.99, 4.75) for births conceived using ovulation drugs, 3.17 (CI 1.4, 7.19) for neonates conceived using IUI and 4.24 (CI 2.05, 8.77) for neonates conceived by IVF, compared to women with subfertility who used no treatment during the month of conception. A reported diagnosis of female factor infertility increased the adjusted odds of having a PTB 2.99 (CI 1.5, 5.97). Duration of pregnancy attempt was not independently associated with PTB. In restricting analyses to singleton gestation, odds ratios were not significant for any type of treatment.

**Conclusion:**

IVF, IUI, and ovulation drugs were all associated with a higher incidence of preterm birth and low birth weight, predominantly related to multiple gestation births.

**Supplementary Information:**

The online version contains supplementary material available at 10.1186/s12978-022-01363-4.

## Plain Language Summary

Infertility treatments such as in vitro fertilization are associated with preterm birth, but less is known about how other less invasive treatments contribute to preterm birth. This study compares different types of fertility treatments and rates of preterm birth with women who are also struggling with infertility but did not use fertility treatments at the time of their pregnancy. 490 women were recruited at the University of Utah between 2010 and 2012. Participants were asked to complete a survey and were linked to birth certificate and fetal death certificate data. Women who used in vitro fertilization were 4.24 times more likely to have a preterm birth than those who used no treatment. Use of intrauterine insemination were 3.17 times more likely to have a preterm birth than those who used no treatment at time of conception. Ovulation stimulating drugs were 2.17 times more likely to have a preterm birth. Having female factor infertility was also associated with higher odds of having preterm birth. For those who are having trouble conceiving, trying less invasive treatments to achieve pregnancy might reduce their risk of preterm birth.

**Supplementary Information:**

The online version contains supplementary material available at 10.1186/s12978-022-01363-4.

## Background

Approximately 9% of couples worldwide experience difficulty conceiving or maintaining a pregnancy; this prolonged duration of non-conception is referred to as subfertility [1–5]. Subfertility is commonly identified as a clinical “infertility” when a couple desiring conception has had regular intercourse without contraception for 12 months or longer without achieving pregnancy [1]. There are varying degrees of subfertility and a variety of potential underlying causes, including abnormalities in oocyte production, sperm production, reproductive tract transport of the sperm, oocyte, and/or embryo, implantation, or other conditions that affect one or multiple components of the reproductive process [6]. Diagnostic tests and tracking menstrual cycle patterns can help to determine the underlying etiology of subfertility [7]. However, frequently providers are unable to identify the precise cause of a couple’s subfertility and 15–30% of couples may be assigned the diagnosis of unexplained infertility [8].

About half of subfertile couples seek medical treatment [5]. Common medical treatments include the use of in vitro fertilization (IVF), intrauterine insemination (IUI), and ovulation stimulation (OS). In the past three decades, the focus of fertility research and treatment has shifted from less invasive medical treatments (including OS and IUI) to more invasive, specifically IVF. IVF was originally developed to overcome absolute subfertility due to blockage or absence of the fallopian tubes, and later expanded to treat severe male subfertility with the addition of intracytoplasmic sperm injection (ICSI) (i.e., specific indications for IVF), but is now frequently used for couples with diminished fertility due to any cause as well as those with infertility of unknown cause [9, 10]. While some advocate that IVF should become a primary management strategy for couples without specific indications because of its high probability of success per cycle success, there are substantial concerns about expanding use of IVF, including high cost and impact on neonatal outcomes [9, 10]. Epidemiologic studies have demonstrated higher incidence of preterm birth (PTB), low birthweight (LBW), and birth defects among children conceived through IVF, when compared to children conceived without medical interventions, even when the analyses are limited to singleton pregnancies [11–13].

In the United States, the Society of Assisted Reproductive Technology (SART) and the Center for Disease Control conduct fertility clinic level surveillance with the goal of tracking IVF procedures and outcomes [14, 15]. The proportion of live births conceived through IVF average 1.4% but vary by region (range 0.2% in Puerto Rico to 4.3% in Massachusetts) [16]. Internationally, IVF and other assisted fertility methods contribute to a considerable proportion of the PTB and LBW infants born each year [17]. No formal surveillance exists for the less invasive treatments, but exposure to these fertility treatments (OS and IUI) may also be associated with adverse perinatal outcomes [18–20]. It is estimated that OS accounts for up to 6% of the births in the United States and IUI for about 1% of births [18, 21]. Monitoring birth outcomes and assessing risks associated with each of these medical exposures are critical public health concerns. Additional questions remain as to whether these adverse outcomes are related to the treatments or to the underlying causes or severity of the subfertility [11, 22]. Few studies exist that assess the independent risks of subfertility [12].

This research aims to provide insight into the relationship between fertility treatments (OS, IUI and IVF) and preterm birth among women with primary subfertility, compared to subfertile women who conceived without fertility treatment. We used data from parallel clinic and population-based cohorts, and examined the contribution of fertility-related diagnoses, as well as the role of multiple gestation.

## Methods

The Fertility Experiences Study (FES) is a retrospective cohort study conducted at the University of Utah between April 2010 and September 2012. Two parallel cohorts were recruited. For the clinic-based cohort, participants were recruited from female patients seen for a new consultation for subfertility and/or treatment at one of the two specialty fertility clinics in Utah between 2000 and 2009. For the population-based cohort, two period-based cohorts were recruited using the Utah Population Database (UPDB) to identify and recruit potentially eligible women of reproductive age range who were married as of 2002 or 2006 but had not yet had a live birth as of the end of 2004 or 2008 (index dates). For both clinic and population-based cohorts, the final eligibility criteria were as follows: Between 20 and 35 years of age at the index date, no pregnancies prior to index date, at least 1 year of regular intercourse without contraception with a male partner at the index date, and a Utah resident during 3 years following the index date. The inclusion of the population cohort allows inclusion of women with subfertility who never receive specialty fertility treatment. Full details of study design and recruitment have been published previously [23]. All participants in the study completed the Fertility Experiences Questionnaires (FEQ), which included a self-administered online questionnaire followed by a structured telephone interview with trained study staff. In comparison to medical records, the FEQ was over 90% sensitive for pregnancy attempt duration, pregnancy outcomes, and use of IVF and IUI, and 70% sensitive for the use of ovulation drugs [24]. Data from 2000 to 2010 Utah birth and fetal death certificates were linked with data from women who completed both the online questionnaire and the structured telephone interview. The University of Utah Institutional Review Board approved this study; participants provided informed consent online prior to completing the initial questionnaire. (University of Utah IRB #27783).

The key exposure measure is the type of fertility treatment received during the month of conception that resulted in the first live birth or fetal death. Treatment groups are defined based on the most invasive medical treatment used during the cycle of conception. For the purposes of this study, the most invasive treatment is IVF. IVF includes all procedures that involve manipulating both sperm and eggs outside the body. The next most invasive treatment was considered to be IUI. Women were categorized as using IUI during the cycle of conception, regardless of if they were also using OS. If women only reported medication to stimulate or enhance ovulation during the conception cycle then they were classified as using OS. Women who did not receive any medication or procedure during the cycle of conception were classified as having no treatment, even if they receive medical treatment during previous cycles, or reported alternative non-medical treatment (such as acupuncture or herbs). This group of untreated subfertile women was used as the control for the analysis.

We assessed the duration of pregnancy attempt, which provides an indicator of severity of subfertility. During the structured telephone interview, trained study staff asked each woman about specific dates when she was at risk for pregnancy. Pregnancy attempt duration was calculated as the interval between the date the participant reported her attempt began and the estimated date of conception. We estimated the date of conception by subtracting the clinical gestational age at birth and date of birth as reported on the birth certificate.

Fertility-related diagnoses were obtained through the self-administered online questionnaire. The question asked “have you or your partner ever been told or suspect that you have any of the following diagnosis?” Answers were yes, no, and unsure. Women who answered “no” or they were “unsure” were considered to have a negative answer. For this analysis, diagnoses were grouped into the Society for Assisted Reproductive Technology Clinical Outcomes Reporting System (SARTCORS) categories. SARTCORS categories are tubal factor, endometriosis, ovulation dysfunction, uterine factor, male factor, or unexplained. If women had more than one female factor diagnosed then they were categorized as multiple female factors. If a couple has a female contributor and a male factor issue then they are categorized as combined male and female factor. For this analysis, any female factor infertility was collapsed into a dichotomous variable, and any male factor was considered a separate dichotomous variable.

The primary outcome measure was preterm birth as determined by gestational age reported on the birth certificate. PTB is defined as any pregnancy that ended in a live birth or fetal death at less than 37 completed weeks of gestation as reported on the state birth certificate [25]. Birth certificate gestational age is typically calculated by the hospital using last menstrual period, confirmed by first trimester ultrasound. The occurrence of multiple gestations was also obtained from the birth certificate or fetal death certificate. In the state of Utah, fetal death certificates are issued for in-utero demise after 20 weeks gestation.

Covariates for the analysis were based on known risk factors for fertility treatments and for preterm birth. Variables considered in the analysis include age of mother at delivery, age of male partner, race, maternal education, prepregnancy BMI, annual income, and religious affiliation. Religious affiliation with the Church of Jesus Christ of Latter-day Saints was identified because of its strong association with avoiding behavioral risk factors for preterm birth, including tobacco use, alcohol use, and drug use [26]. Maternal and paternal age and maternal BMI were obtained from the birth certificate. Parental age was categorized as less than 30 years or 30 years old or older at the time of delivery. BMI was calculated using prepregnancy weight and height and dichotomized as underweight/normal BMI (< 25 kg/m^2^) and Overweight/Obese BMI (≥ 25 kg/m^2^). While low-BMI is a known risk factor for PTB we collapsed with normal BMI as numbers of low-BMI participants were insufficient (n = 21), we also address this in the sensitivity analysis. Education, income, and LDS religious affiliation were obtained from the FEQ survey. Education was dichotomized as less than college graduation and college graduation or more. Income was grouped as annual household income of less than $50,000, $50,000–$99,000, and $100,000.

The frequency of PTB was compared across participant characteristics and exposure variables. Crude and adjusted odds ratios and 95% confidence intervals for each exposure (treatment category) and each birth outcome were estimated using simple and multivariable logistic regression. Parental age, prepregnancy BMI, education, income, and LDS religious affiliation, and treatment received during the cycle of conception were included as potential confounders in the base model. Subsequent models assessed additional potential confounders including extended duration of pregnancy attempt (less than 24 months vs 24 months or more), self-reported diagnosis categories (female and male factor categories), and multiple gestation dichotomized. We repeated the analyses restricted to singleton births. Data were analyzed using Stata14 or higher (College Station, TX).

A sensitivity analysis was conducted using BMI, and duration of attempt as continuous variables. We performed a logistic regression removing participants who reported a fertility related diagnosis related to tubal factors, as these women would not have been able to conceive without treatment by IVF. Additional sensitivity analysis was conducted for women who were subfertile using the screening question but may have had intervening breaks in their pregnancy attempt (due to birth control for personal or medical use, miscarriage, or other reasons) so that their cumulative time at risk of pregnancy was found to be less than 12 months. A flow diagram describing exclusion and loss to follow up has been published previously [23].

## Results

Study participants reported a total of 492 first births in the FEQ telephone interview. Of these, 491 were linked to state vital records—488 came from birth certificates and 3 came from fetal death records. One participant was excluded from the analysis due to an unintended pregnancy that occurred while not actively trying to get pregnant. There were some notable differences in attempt duration, treatment and diagnosis patterns between individuals recruited from the population cohort and those who had been recruited for the general population. Specifically, in the population cohort, over half 53.5% had had a live birth by the time we interviewed them for the study, close to a third (31.3%) had never received any type of fertility treatment, 5% reported trying alternative treatments, 29% had used fertility drugs, 20% had used artificial insemination, and 14% had used IVF. Among the participants from the clinic cohort, 58% had had a live birth by the time we interviewed them for the study, 6.5% had never received any type of fertility treatment and 1.7% reported trying alternative treatments, 15.9% had used fertility drugs, 29% had used artificial insemination, and 47.3% had used IVF. Of the 490 linked live births while intending to get pregnant, 19% were preterm. Table 1 displays the distribution of maternal characteristics and demographics by PTB outcomes.

**Table 1:** Characteristics of women by preterm birth (< 37 weeks gestational age)

	Term (> 37 weeks)		Preterm (< 37)		Total		P value
	N	Row%	N	Row%	N	Col%	
Maternal age at delivery							
≤ 30	290	81.7%	65	18.3%	355	72.4%	0.669
31+	108	80.0%	27	20.0%	135	27.6%	
Paternal age at delivery							
≤ 30	239	83.0%	49	17.0%	288	58.9%	0.223
31+	158	78.6%	43	21.4%	201	41.1%	
BMI category (pre-pregnancy)							
Underweight/Normal	184	76.0%	58	24.0%	242	55.1%	0.148
Overweight/Obese	161	81.7%	36	18.3%	197	4.9%	
Income (at interview)							
Less than $50,000	110	81.5%	25	18.5%	135	28.7%	0.938
50,000–$99,999	213	80.1%	53	19.9%	266	56.6%	
Over $100,000	56	81.2%	13	18.8%	69	14.7%	
Education level (at interview)							
Less than college grad	127	77.4%	37	22.6%	164	33.6%	0.136
College grad or more	269	83.0%	55	17.0%	324	66.4%	
Race/Ethnicity							
White, non-Hispanic	381	80.9%	90	19.1%	471	96.1%	0.348
Hispanic, other non-White	17	89.5%	2	10.5%	19	3.9%	
Religion							
Non-LDS	84	78.5%	23	21.5%	107	21.8%	0.415
Latter-day Saint	314	82.0%	69	18.0%	383	78.2%	
Attempt duration to conception							
< 12 months	69	81.2%	16	18.8%	85	17.4%	0.771
12 to < 24	100	81.3%	23	18.7%	123	25.2%	
24 to < 36	73	85.9%	12	14.1%	85	17.4%	
36 to < 48	58	79.5%	15	20.5%	73	14.9%	
48+	97	78.9%	26	21.1%	123	25.2%	
Recruitment cohort							
Clinic	203	76.3%	63	23.7%	266	54.3%	0.002
Population	195	87.1%	29	12.9%	224	45.7%	
Baby sex							
F	188	79.0%	50	21.0%	238	48.6%	0.219
M	210	83.3%	42	16.7%	252	51.4%	
Multiplicity							
Singleton	374	90.8%	38	9.2%	412	84.1%	0.000
Twins	24	33.8%	47	66.2%	71	14.5%	
Triplets	0	0.0%	7	100.0%	7	1.4%	
Treatment in cycle of conception							
None	190	89.2%	23	10.8%	214	43.5%	0.001
Drugs	61	79.2%	16	20.8%	77	15.7%	
IUI	48	75.0%	16	25.0%	64	13.1%	
IVF	99	72.8%	37	27.2%	136	27.8%	
Total	398	81.2%	92	18.8%	490	100.0%	

In the 490 subfertile women, 41% reported having unexplained infertility, 40% reported male factor infertility, 54% reported a diagnosis of ovulation dysfunction, 27% endometriosis, 16% a tubal factor, 13% uterine factor infertility, 28% multiple female factors, and 12% blocked or damaged fallopian tubes (not mutually exclusive). Overall during the cycle of conception, 44% had no infertility treatment, 16% used OS, 13% had IUI, and 28% had IVF (Table 2). Of the 13% that used IUI, 99% also used ovulation drugs (OS). Types of treatments used during the cycle of conception were similar among women who reported tubal factor, endometriosis, or unexplained infertility. However, women who reported ovulatory dysfunction more often reported OS medication (24% vs. 16% for all women); women with uterine factor more frequently reported IVF (40% vs. 28% for all women); and women with unexplained infertility most commonly reported not using any treatment during the cycle of conception (47% vs. < 38% for all other categories; Table 2). Women with the following diagnoses had a higher incidence of PTB than women without the respective diagnosis: tubal factor (27% vs 17%); multiple female factors diagnosis (25% vs 17%); endometriosis (24% vs 17%; Table 3).

**Table 2:** Most invasive treatment during the conception cycle by infertility diagnosis (N = 490)

	None		Drugs		IUI*		IVF		Total		P value
	N	Row%	N	Row%	N	Row%	N	Row%	N	Col%	
Tubal factor	29	37.2%	14	17.9%	9	11.5%	26	33.3%	78	15.9%	0.550
Endometriosis	49	37.4%	18	13.7%	19	14.5%	45	34.4%	131	26.7%	0.169
Ovulation dysfunction	99	37.5%	63	23.9%	41	15.5%	61	23.1%	264	53.9%	0.000
Uterine factor	20	30.8%	7	10.8%	12	18.5%	26	40.0%	65	13.3%	0.022
Male factor	60	30.8%	21	10.8%	27	13.8%	87	44.6%	195	39.8%	0.000
Unexplained infertility	136	47.4%	43	15.0%	32	11.1%	76	26.5%	287	59.5%	0.176
Multiple female factors	45	32.6%	26	18.8%	25	18.1%	42	30.4%	138	28.2%	0.014
Multiple female and male	43	33.1%	19	14.6%	16	12.3%	52	40.0%	130	26.5%	0.002
Total	213	43.5%	77	15.7%	64	13.1%	136	27.8%	490	100.0%	

Women/couples may be in more than one category

*99% of IUI cycles also had ovulation drugs

**Table 3:** Birth outcomes by infertility diagnosis (N = 490)

	Term		Preterm		Total		P value*
	N	Row%	N	Row%	N	Col%	
Tubal factor							
No	341	82.8%	71	17.2%	412	84.1%	0.044
Yes	57	73.1%	21	26.9%	78	15.9%	
Endometriosis							
No	299	83.3%	60	16.7%	359	73.3%	0.053
Yes	99	75.6%	32	24.4%	131	26.7%	
Ovulation dysfunction							
No	190	84.1%	36	15.9%	226	46.1%	0.135
Yes	208	78.8%	56	21.2%	264	53.9%	
Uterine factor							
No	347	81.6%	78	18.4%	425	86.7%	0.540
Yes	51	78.5%	14	21.5%	65	13.3%	
Male factor							
No	243	82.4%	52	17.6%	295	60.2%	0.423
Yes	155	79.5%	40	20.5%	195	39.8%	
Unexplained infertility							
No	235	81.9%	52	18.1%	287	59.5%	0.604
Yes	156	80.0%	39	20.0%	195	40.5%	
Multiple female factors							
No	294	83.5%	58	16.5%	352	71.8%	0.037
Yes	104	75.4%	34	24.6%	138	28.2%	
Multiple female and male factors							
No	299	83.1%	61	16.9%	360	73.5%	0.084
Yes	99	76.2%	31	23.8%	130	26.5%	
Total	398	81.2%	92	18.8%	490	100.0%	

*χ^2^ comparing to women who were not told or suspect diagnosis

∙ Diagnostic categories—SART CORS classification

– Tubal factor—pelvic adhesion or scarring, blocked or damaged fallopian tubes

– Endometriosis

– Ovulation dysfunction—low progesterone, low estrogen, not ovulating, abnormal ovulation, lutenized unruptured follicule (LUF), Luteal Phase Defect (LUD), PCOS

– Uterine factor—hostile or limited cervical mucus, fibroids in the uterus, polyps in the uterus,

– Male factor

– Unknown infertility—unexplained subfertility

– Multiple female factors—more than one of the following diagnosis Tubal, Endometriosis, Ovulation dysfunction, or Uterine

– Female and male factor—male factor plus at least one female factor

Many women reported use of more invasive treatments outside the cycle of conception. For example, of women who conceived using no treatment, 15% had tried OS previously, 17% had tried IUI, and 16% had tried IVF (see Additional file 1: Table S1).

Each type of treatment used during the cycle of conception were associated with increased odds of PTB in the unadjusted model when compared to women that conceived spontaneously. The odds of PTB increased with increased invasiveness of treatment in both unadjusted and adjusted analyses. After adjustment for maternal age, paternal age, maternal education, annual income, religious affiliation, female or male fertility diagnosis, and duration of subfertility, the odds of having a PTB were 2.17 times higher (95% CI 0.99, 4.75) for women who conceived using ovulation drugs, 3.17 times higher (95% CI 1.40, 7.19) for women who conceived using IUI and 4.24 times higher (95% CI 2.05, 8.77) for women who conceived by IVF, compared to women with subfertility who used no treatment during the month of conception. Duration of pregnancy attempt was not independently associated with PTB. A reported diagnosis of female factor infertility increased the adjusted odds of having a PTB was 2.99 times higher (95% CI 1.50, 5.97) compared to women who did not report any female factor infertility. In sensitivity analyses excluding women with tubal factor infertility, the odds of PTB were about the same for most invasive treatment during cycle of conception, and still significant (aOR 2.75, 95% CI 1.42, 5.31) for women with any female factor infertility. Only 6.6% of the births conceived without any treatment during the month of conception were twins, for OS this increased to 19% twins and 6% triplets, IUI births were 10.9% twins and 5% triplets, and IVF births were 30% twins and 2% triplets. Accordingly, when multiple gestation was added to the model, it had the highest association with PTB (aOR 28.0 95% CI 15.60, 68.60). Table 4 details the results from the unadjusted and adjusted logistic regression models.

**Table 4:** Unadjusted and adjusted odds of preterm birth for most invasive treatment in the cycle of conception

	Unadjusted OR [95% CI]	Adjusted OR [95% CI]	Adjusted OR, with adjustment for multiple gestation [95% CI]
Most invasive treatment used during cycle of conception			
None	Reference	Reference	Reference
Drugs	2.17 (1.08, 4.36)	2.17 (0.99, 4.75)	1.34 (0.52, 3.45)
IUI	2.75 (1.35, 5.61)	3.17 (1.4, 7.19)	2.16 (0.82, 5.69)
IVF	3.09 (1.74, 5.48)	4.24 (2.05, 8.77)	1.46 (0.59, 3.58)
Etiology			
No female factor		Reference	Reference
Any female factor		2.99 (1.5, 5.97)	3.00 (1.32, 6.79)
No male factor		Reference	Reference
Any male factor		1.01 (0.58, 1.76)	0.99 (0.51, 1.95)
Multiple gestation			
Singleton			Reference
Multiple			27.91 (13.25, 58.79)
Maternal age at delivery			
≤ 30		Reference	Reference
31+		1.03 (0.5, 2.09)	0.91 (0.38, 2.19)
Paternal age at delivery			
≤ 30		Reference	Reference
31+		1.54 (0.8, 2.97)	1.42 (0.64, 3.13)
BMI category (at delivery)			
Underweight/Normal		Reference	Reference
Overweight/Obese		0.9 (0.52, 1.55)	1.19 (0.61, 2.33)
Income			
Less than $50,000		Reference	Reference
50,000–$99,999		1.13 (0.6, 2.14)	0.81 (0.38, 1.72)
Over $100,000		0.71 (0.28, 1.84)	0.85 (0.27, 2.73)
Education level			
Less than college grad		Reference	Reference
College grad or more		0.7 (0.4, 1.23)	0.84 (0.42, 1.67)
Religion			
Non-LDS		Reference	Reference
Latter-day Saint		0.87 (0.45, 1.67)	0.73 (0.32, 1.66)
Attempt duration ending in conception			
< 24		Reference	Reference
≥ 24		0.59 (0.33, 1.06)	0.66 (0.33, 1.34)

## Discussion

When compared to subfertile women who did not use any fertility treatments during the cycle of conception, women who used any kind of fertility treatment were more significantly more likely to deliver preterm. As the invasiveness of treatment increased, so did both the incidence of multiple gestation and the incidence of PTB. Women who used OS to conceive were more than twice as likely to deliver preterm compared to women who used no treatment, while women who used IVF were about four times as likely. A large body of research has previously established this relationship for IVF, and some studies have also found it for IUI [11, 19, 22, 27, 28]. The level of invasiveness of treatment may have a direct impact on PTB and/or it may be a marker for level of severity of underlying subfertility [29, 30]. The association was very closely related to the incidence of multiple gestation, which is not a confounding factor for the relationship between treatment and PTB, but an intermediary in the pathway between treatment and outcome (PTB) [18, 31]. Thus, this research is consistent with a large body of research showing that the predominant factor linking PTB to fertility treatment is multiple gestation [32, 33]. However, recent population-based research has indicated an association of IVF with PTB among singletons [12, 34–36]. Of additional note, this risk of PTB across the subfertile cohort, including the subfertile controls was markedly higher than population rates of PTB. This points to a relationship between the underlying etiology as well as medical interventions as risk factors for PTB. Our study did not have sufficient sample size to detect a smaller impact among singletons. However, we did find an independent association between female factor infertility etiology and PTB.

Few studies have compared birth outcomes of subfertile women conceiving with fertility treatments with subfertile women who conceive spontaneously [12]. Our population-based sampling captured subfertile women who never sought treatment, or who only had treatment outside of specialty fertility clinics, allowing for a much more population-relevant perspective of the impact of fertility treatment [23]. The use of an untreated subfertile population as the referent category for a variety of treatment exposures is a strength of this study and may present a treatment effect magnitude that at least partially controls for misclassification of fertility related diagnosis and undiagnosed subfertility pathology.

We validated our questionnaire for the woman’s report of treatment [24]; other research in the United States has also found high correlation between women’s self-reported treatment and that found in medical records [37]. The validity of self-reported fertility diagnosis is less certain, but at least some types of diagnoses have been found to be reported accurately in questionnaires by educated women [38, 39]. We sought to minimize problems with recall for treatment by the multimode, two stage questionnaire [24].

Generalizability of findings may also be limited by the geographic location with a relatively homogenous racial and ethnic population, and a relatively lower prevalence of smoking, alcohol, and drug use. However, this population may also for a more direct effect of the effect of treatment to be evaluated, as Utah was noted to have the highest proportion of women giving birth from fertility treatments of 38 states examined from birth certificate data (about 5% of births across all types of medical treatment) [40]. Additionally, there are some limitations in the accuracy of gestational age from birth certificates, but these clinically relevant estimates are typically confirmed with early ultrasound [41]. We did not distinguish between spontaneous labor or iatrogenic labor for women delivering at less than 37 weeks’ gestation: in future studies we recommend that this is taken into account [42].

PTB is a significant public health issue worldwide. In the United States, more than 11% of live born infant are born at gestational ages < 37 weeks. PTB contributes largely to infant and child morbidity and mortality [43, 44]. More than 26.2 billion dollars are spent in the United States each year on costs associated with PTB [25]. The findings from this analysis support the proposition that all medical fertility treatments contribute directly to the incidence of PTB, principally by increasing multiple births. Efforts should be made to reduce the incidence of multiple gestation from all fertility treatments, not just IVF [45–47]. However, based on these and other data, we cannot exclude the possibility that even if all multiple gestations are eliminated, there may remain some risk for preterm birth among singletons [12]. Thus, we support the need for more rigorous population surveillance on the use of all fertility treatments, not just IVF [48]. While treatment patterns may have changed since data collection, the findings remain relevant to current practices and support additional investigation of ways fertility interventions are driving current PTB rates.

We suggest that individuals who are experiencing difficulty conceiving should consider first the opportunity for conception with less invasive treatments or no treatment. Although the time to conception may be longer, the potential for improved optimal birth outcomes should be weighed strongly against the desire to conceive faster. Additional research needs to be conducted to assess time to live birth in subfertile populations using a variety of fertility treatments [49]. The risk of PTB after conception using OS or IUI is increased on an even greater magnitude to smoking, yet clinicians and patients may pay less attention to the risk of treatment [50, 51].

## Conclusions

Our findings support efforts to encourage women to give an adequate trial of the least invasive fertility treatment that may work for them, and to modify the practice of all fertility treatments to minimize incidence of multiple gestation. Future research should consider interventions that may prevent preterm birth among these higher risk populations of subfertile women, regardless of type of treatment received.

## Supplementary Information

**Additional file 1: Table S1.** Most invasive treatment used during the month of conception and ever used.

## Data Availability

Data used for this analysis is available from the corresponding author on reasonable request.

## Abbreviations

IUI: Intrauterine insemination
PTB: Preterm birth
CI: Confidence intervals
IVF: In vitro fertilization
OS: Ovulation stimulation
ICSI: Intracytoplasmic sperm injection
LBW: Low birthweight
SART: Society of Assisted Reproductive Technology
FES: Fertility Experiences Study
UPDB: Utah Population Database
FEQ: Fertility Experiences Questionnaire
IRB: Institutional Review Board
SARTCORS: Society for Assisted Reproductive Technology Clinical Outcomes Reporting System
BMI: Body Mass Index
LDS: Church of Jesus Christ of Latter-day Saints
aOR: Adjusted odds ratio

## References

[1] Thoma ME, McLain AC, Louis JF, King RB, Trumble AC, Sundaram R (2013). Prevalence of infertility in the United States as estimated by the current duration approach and a traditional constructed approach. Fertil Steril.

[2] Maconochie N, Doyle P, Prior S (2004). The National Women's Health Study: assembly and description of a population-based reproductive cohort. BMC Public Health.

[3] Keiding N, Kvist K, Hartvig H, Tvede M, Juul S (2002). Estimating time to pregnancy from current durations in a cross-sectional sample. Biostatistics.

[4] Gnoth C, Godehardt E, Frank-Herrmann P, Friol K, Tigges J, Freundl G (2005). Definition and prevalence of subfertility and infertility. Hum Reprod (Oxford, England).

[5] Boivin J, Bunting L, Collins JA, Nygren KG (2007). International estimates of infertility prevalence and treatment-seeking: potential need and demand for infertility medical care. Hum Reprod (Oxford, England).

[6] Strauss JF, Barbieri RL. Yen and Jaffe's reproductive endocrinology: physiology, pathophysiology, and clinical management. 6th ed. Philadelphia: Saunders/Elsevier; 2009. xv, 928 p.

[7] Stanford JB, Parnell TA, Boyle PC (2008). Outcomes from treatment of infertility with natural procreative technology in an Irish general practice. J Am Board Fam Med.

[8] Practice Committee of the American Society for Reproductive Medicine. Effectiveness and treatment for unexplained infertility. Fertil Steril. 2006;86(5 Suppl 1):S111–4. 10.1016/j.fertnstert.2006.07.1475 17055802

[9] Reindollar RH, Regan MM, Neumann PJ, Levine BS, Thornton KL, Alper MM (2010). A randomized clinical trial to evaluate optimal treatment for unexplained infertility: the fast track and standard treatment (FASTT) trial. Fertil Steril.

[10] Welmerink DB, Voigt LF, Daling JR, Mueller BA (2010). Infertility treatment use in relation to selected adverse birth outcomes. Fertil Steril.

[11] Wisborg K, Ingerslev HJ, Henriksen TB (2010). In vitro fertilization and preterm delivery, low birth weight, and admission to the neonatal intensive care unit: a prospective follow-up study. Fertil Steril.

[12] Declercq E, Luke B, Belanoff C, Cabral H, Diop H, Gopal D (2015). Perinatal outcomes associated with assisted reproductive technology: the Massachusetts Outcomes Study of Assisted Reproductive Technologies (MOSART). Fertil Steril.

[13] Barnhart KT (2013). Assisted reproductive technologies and perinatal morbidity: interrogating the association. Fertil Steril.

[14] Zegers-Hochschild F, Adamson GD, de Mouzon J, Ishihara O, Mansour R, Nygren K (2009). The International Committee for Monitoring Assisted Reproductive Technology (ICMART) and the World Health Organization (WHO) revised glossary on ART terminology, 2009. Hum Reprod (Oxford, England).

[15] Centers for Disease Control and Prevention. National public health action plan for the detection, prevention, and management of infertility. Atlanta: Centers for Disease Control and Prevention; 2014.

[16] Sunderam S, Kissin DM, Flowers L, Anderson JE, Folger SG, Jamieson DJ (2012). Assisted reproductive technology surveillance—United States, 2009. Morb Mortal Wkly Rep Recomm Rep.

[17] Kushnir VA, Barad DH, Albertini DF, Darmon SK, Gleicher N (2017). Systematic review of worldwide trends in assisted reproductive technology 2004–2013. Reprod Biol Endocrinol.

[18] Schieve LA, Devine O, Boyle CA, Petrini JR, Warner L (2009). Estimation of the contribution of non-assisted reproductive technology ovulation stimulation fertility treatments to US singleton and multiple births. Am J Epidemiol.

[19] Messerlian C, Platt RW, Tan SL, Gagnon R, Basso O. Low-technology assisted reproduction and the risk of preterm birth in a hospital-based cohort. Fertil Steril. 2015;103(1):81–8 e2. 10.1016/j.fertnstert.2014.10.006 25456793

[20] Messerlian C, Maclagan L, Basso O (2013). Infertility and the risk of adverse pregnancy outcomes: a systematic review and meta-analysis. Hum Reprod (Oxford, England).

[21] Simonsen SE, Baksh L, Stanford JB (2012). Infertility treatment in a population-based sample: 2004–2005. Matern Child Health J.

[22] Jaques AM, Amor DJ, Baker HW, Healy DL, Ukoumunne OC, Breheny S (2010). Adverse obstetric and perinatal outcomes in subfertile women conceiving without assisted reproductive technologies. Fertil Steril.

[23] Stanford JB, Sanders JN, Simonsen SE, Hammoud A, Gibson M, Smith KR (2016). Methods for a retrospective population-based and clinic-based subfertility cohort study: the fertility experiences study. Paediatr Perinat Epidemiol.

[24] Thomas FS, Stanford JB, Sanders JN, Gurtcheff SE, Gibson M, Porucznik CA (2015). Development and initial validation of a fertility experiences questionnaire. Reprod Health.

[25] McCormick MC, Behrman RE (2007). The quiet epidemic of premature birth: commentary on a recent Institute of Medicine report. Ambul Pediatr.

[26] Daniels M, Merrill RM, Lyon JL, Stanford JB, White GL (2004). Associations between breast cancer risk factors and religious practices in Utah. Prev Med.

[27] Malchau SS, Loft A, Henningsen AK, Nyboe Andersen A, Pinborg A. Perinatal outcomes in 6,338 singletons born after intrauterine insemination in Denmark, 2007 to 2012: the influence of ovarian stimulation. Fertil Steril. 2014;102(4):1110–6 e2. 10.1016/j.fertnstert.2014.06.034 25064412

[28] Poon WB, Lian WB (2013). Perinatal outcomes of intrauterine insemination/clomiphene pregnancies represent an intermediate risk group compared with in vitro fertilisation/intracytoplasmic sperm injection and naturally conceived pregnancies. J Paediatr Child Health.

[29] Stanford JB, Simonsen SE, Baksh L (2016). Fertility treatments and adverse perinatal outcomes in a population-based sampling of births in Florida, Maryland, and Utah: a cross-sectional study. BJOG.

[30] Luke B, Stern JE, Hornstein MD, Kotelchuck M, Diop H, Cabral H (2016). Is the wrong question being asked in infertility research?. J Assist Reprod Genet.

[31] Diamond MP, Legro RS, Coutifaris C, Alvero R, Robinson RD, Casson P (2015). Letrozole, gonadotropin, or clomiphene for unexplained infertility. N Engl J Med.

[32] Sunderam S, Kissin DM, Crawford SB, Folger SG, Boulet SL, Warner L (2018). Assisted reproductive technology surveillance—United States, 2015. Morb Mortal Wkly Rep Recomm Rep.

[33] Schieve LA, Meikle SF, Ferre C, Peterson HB, Jeng G, Wilcox LS (2002). Low and very low birth weight in infants conceived with use of assisted reproductive technology. N Engl J Med.

[34] Stern JE, Liu C-L, Cabral HJ, Richards EG, Coddington CC, Hwang S (2018). Birth outcomes of singleton vaginal deliveries to ART-treated, subfertile, and fertile primiparous women. J Assist Reprod Genet.

[35] Hwang SS, Dukhovny D, Gopal D, Cabral H, Missmer S, Diop H, et al. Health of infants after ART-treated, subfertile, and fertile deliveries. Pediatrics. 2018;142(2):e20174069. 10.1542/peds.2017-4069 PMC6317642 29970386

[36] Luke B, Brown MB, Wantman E, Seifer DB, Sparks AT, Lin PC (2019). Risk of prematurity and infant morbidity and mortality by maternal fertility status and plurality. J Assist Reprod Genet.

[37] Buck Louis GM, Hediger ML, Bell EM, Kus CA, Sundaram R, McLain AC (2014). Methodology for establishing a population-based birth cohort focusing on couple fertility and children's development, the Upstate KIDS Study. Paediatr Perinat Epidemiol.

[38] Saha R, Marions L, Tornvall P. Validity of self-reported endometriosis and endometriosis-related questions in a Swedish female twin cohort. Fertil Steril. 2017;107(1):174–8 e2. 10.1016/j.fertnstert.2016.09.038 27793372

[39] Rich-Edwards JW, Goldman MB, Willett WC, Hunter DJ, Stampfer MJ, Colditz GA (1994). Adolescent body mass index and infertility caused by ovulatory disorder. Am J Obstet Gynecol.

[40] Osterman MJ, Martin JA, Curtin SC, Matthews TJ, Wilson EC, Kirmeyer S. Newly released data from the revised U.S. birth certificate, 2011. Natl Vital Stat Rep. 2013;62(4):1–22. 24351136

[41] Andrade SE, Scott PE, Davis RL, Li DK, Getahun D, Cheetham TC (2013). Validity of health plan and birth certificate data for pregnancy research. Pharmacoepidemiol Drug Saf.

[42] Simonsen SE, Lyon JL, Stanford JB, Porucznik CA, Esplin MS, Varner MW (2013). Risk factors for recurrent preterm birth in multiparous Utah women: a historical cohort study. BJOG.

[43] Swamy GK, Ostbye T, Skjaerven R (2008). Association of preterm birth with long-term survival, reproduction, and next-generation preterm birth. JAMA.

[44] Kramer MS, Demissie K, Yang H, Platt RW, Sauve R, Liston R. The contribution of mild and moderate preterm birth to infant mortality. Fetal and Infant Health Study Group of the Canadian Perinatal Surveillance System. JAMA. 2000;284(7):843–9. 10.1001/jama.284.7.843 10938173

[45] Kulkarni AD, Jamieson DJ, Jones HW, Kissin DM, Gallo MF, Macaluso M (2013). Fertility treatments and multiple births in the United States. N Engl J Med.

[46] Allen BD, Adashi EY, Jones HW (2014). On the cost and prevention of iatrogenic multiple pregnancies. Reprod Biomed Online.

[47] Stanford JB (2017). What kind of policies for fertility treatment would improve affordability and outcomes for individuals and the public?. Paediatr Perinat Epidemiol.

[48] Stanford JB, Martin JC, Gibson M, Birdsall E, Brixner DI (2013). Use of clomiphene citrate in the University of Utah Community Clinics. J Reprod Med.

[49] Stanford JB, Mikolajczyk RT, Lynch CD, Simonsen SE (2010). Cumulative pregnancy probabilities among couples with subfertility: effects of varying treatments. Fertil Steril.

[50] Practice Committee of the American Society for Reproductive Medicine. Use of clomiphene citrate in infertile women: a committee opinion. Fertil Steril. 2013;100(2):341–8. 10.1016/j.fertnstert.2013.05.033 23809505

[51] Kolas T, Nakling J, Salvesen KA (2000). Smoking during pregnancy increases the risk of preterm births among parous women. Acta Obstet Gynecol Scand.

