# Supplementary material for: Fertility treatments and the risk of preterm birth among women with subfertility: a linked-data retrospective cohort study
Source: Reprod Health. 2022 Mar 29;19:83. doi: 10.1186/s12978-022-01363-4 (PMC8966354; doi:10.1186/s12978-022-01363-4)
Supplement: Supplementary file 1 — Additional file 1: Table S1. Most invasive treatment used during the month of conception and ever used. [file 12978_2022_1363_MOESM1_ESM.docx]

***Additional Table S1. Most invasive treatment used during the month of conception and ever used***

|  | Most invasive treatment ever used | | | | |  |
| --- | --- | --- | --- | --- | --- | --- |
| Most invasive treatment during conception cycle |  | None  n (%) | Drugs  n (%) | IUI  n (%) | IVF  n (%) | **Total** |
|  | None | 113 (52%) | 33 (15%) | 36 (17%) | 35 (16%) | **217** |
|  | Drugs | 0 (0%) | 59 (81%) | 9 (12%) | 5 (7%) | **73** |
|  | IUI | 0 (0%) | 0 (0%) | 60 (94%) | 4 (6%) | **64** |
|  | IVF | 0 (0%) | 0 (0%) | 0 (0%) | 136 (100%) | **136** |
|  | ***Total*** | ***113*** | ***92*** | ***105*** | ***180*** | ***490*** |
